# Combining Evolutionary and Adaptive Control Strategies for Quadruped Robotic Locomotion

**DOI:** 10.3389/fnbot.2019.00071

**Published:** 2019-08-29

**Authors:** Elisa Massi, Lorenzo Vannucci, Ugo Albanese, Marie Claire Capolei, Alexander Vandesompele, Gabriel Urbain, Angelo Maria Sabatini, Joni Dambre, Cecilia Laschi, Silvia Tolu, Egidio Falotico

**Affiliations:** ^1^The BioRobotics Institute, Scuola Superiore Sant'Anna, Pontedera, Italy; ^2^Automation and Control Group, Department of Electrical Engineering, Technical University of Denmark, Copenhagen, Denmark; ^3^AIRO, Electronics and Information Systems Department, Ghent University - imec, Ghent, Belgium

**Keywords:** evolutionary algorithm, bio-inspired controller, cerebellum-inspired algorithm, robotic locomotion, neurorobotics, central pattern generator

## Abstract

In traditional robotics, model-based controllers are usually needed in order to bring a robotic plant to the next desired state, but they present critical issues when the dimensionality of the control problem increases and disturbances from the external environment affect the system behavior, in particular during locomotion tasks. It is generally accepted that the motion control of quadruped animals is performed by neural circuits located in the spinal cord that act as a Central Pattern Generator and can generate appropriate locomotion patterns. This is thought to be the result of evolutionary processes that have optimized this network. On top of this, fine motor control is learned during the lifetime of the animal thanks to the plastic connections of the cerebellum that provide descending corrective inputs. This research aims at understanding and identifying the possible advantages of using learning during an evolution-inspired optimization for finding the best locomotion patterns in a robotic locomotion task. Accordingly, we propose a comparative study between two bio-inspired control architectures for quadruped legged robots where learning takes place either during the evolutionary search or only after that. The evolutionary process is carried out in a simulated environment, on a quadruped legged robot. To verify the possibility of overcoming the reality gap, the performance of both systems has been analyzed by changing the robot dynamics and its interaction with the external environment. Results show better performance metrics for the robotic agent whose locomotion method has been discovered by applying the adaptive module during the evolutionary exploration for the locomotion trajectories. Even when the motion dynamics and the interaction with the environment is altered, the locomotion patterns found on the learning robotic system are more stable, both in the joint and in the task space.

## 1. Introduction

From the outside, locomotion appears to be performed spontaneously and effortlessly by both animals and humans, but a complex neural system controls it. Movements are mainly controlled by the Central Nervous System (CNS) which generates commands at a cortical and spinal level and integrate those commands based on different sensory feedback. All the muscular activation and coordination processes can be unexpectedly produced without the need for conscious control (Takakusaki, 2013). In quadrupeds, the neural control of locomotion happens along with all the CNS, involving the contribution of cortical areas as the pre-motor and motor cortices and also more peripheral areas such as the spinal cord. In particular, the existence of a Central Pattern Generator (CPG) in the spinal cord has been first demonstrated in the middle of the twentieth century (Hughes and Wiersma, 1960). It is a network of cells that generates basic locomotion patterns by the repetitive contraction of different muscle groups thanks to its periodic oscillations in exciting or inhibiting certain motoneurons.

The cerebellum plays an important role, too, in both quadruped and human locomotion. It improves the accuracy in motor learning, adaptation and cognition on the control commands from the motor cortex (Ito, 2000), computing the inverse dynamics of a body component and delivering a contribution to the present neural signals from the motor cortex (Kawato and Gomi, 1992; Wolpert et al., 1998). In nature, the optimal locomotion strategies are discovered by the long process of evolution. Evolution bases its research on a no-random selection of randomly generated individuals and the final evaluation strictly depends on the agent and its interaction with the surrounding environment. By inspiration from the biological evolution process, the new concept called *Embodied intelligence* or *Embodied brain* emerged more recently (Starzyk, 2008). The idea conveys the importance of the body to properly learn the interaction between intelligence and outer world. Evolution and learning operate on different time scales but both are forms of biological adaptation from which is important to take inspiration from. Evolution reacts to slow environmental changes whereas learning produces adaptive reactions in an individual during its lifetime (Pratihar, 2003).

In robotics, finding effective locomotion strategies has always been a challenge and this task gets even more complicated when the environmental conditions change. To face dynamical external conditions, different methods have been developed, in robotics, and leg-based motion is one of the most effective locomotion mechanism to deal with changing terrains (Full and Koditschek, 1999). However, legged locomotion is usually very complex to be modeled and controlled due to the high-dimensional, nonlinear and dynamically coupled interactions between the robot and the environment. New approaches, employing synergies and symmetries, have been proposed to simplify the problem and decrease its redundancy (Ijspeert, 2008). In some cases, bio-inspired CPG-based controllers have been used to prove how a primitive neural circuit used for generating periodic motion patterns can be extended for generating different types of locomotion. For instance, the research work from Ijspeert et al. (2007) shows a CPG model which switches between swimming-like to walking-like locomotion by just changing a few parameters of the model, as the oscillation threshold of the system.

The need for refined motor control pushed bio-inspired robotics to deeply study the cerebellar contribution and design mathematical models to mimic some of its biological functions in motion control (Wolpert et al., 1998). Cerebellar-like neuro-controllers have also been implemented recently. The cerebellum exploits long-term synaptic plasticity (LTP) to store information about body-object dynamics and to generate internal models of movements. This evidence has been studied by Garrido Alcazar et al. (2013) and implemented for adaptable gain control for robotic manipulation tasks. In this case, it is useful to have cerebellar corrective torques which are self-adaptable, operate over multiple time scales and improve learning accuracy, in order to minimize the motor error. An error-dependent signal operating as a teaching contribution is needed for this purpose.

The interesting interaction between CPG-based oscillators and cerebellar inspired networks has been implemented in bio-inspired control design, too. In the research work proposed by Fujiki et al. (2015), the spinal model generates rhythmic motor commands using an oscillator network based on a Central Pattern Generator and modulates the commands formulated in immediate response to foot contact, while the cerebellar model modifies motor commands, through learning, based on error information related to the difference between the predicted and the actual foot contact timings of each leg.

Another interesting research branch is *evolutionary robotics* which is becoming a very popular approach in the search for new robotic morphology and controllers. The main advantage of this approach is that it is “*prejudice-free,”* in the sense that it mainly depends on the behavior of an agent in interaction with the external environment. In fact, genetic algorithms derive from the kind of long-term adaptation that humans share with other species. This idea of adaptation is meant as a relational property that involves the agent, its environment, and the maintenance of some constraints and can be in the wide sense described as the ability of an agent of interacting with its environment to maintain some existence constraints. Thus, the idea is exploiting the sensorimotor interactions with a dynamic environment to minimize the prior assumptions that are built into a “human-made” model, which reduces the capability of the model itself to count for new and unknown relevant features or artifacts in the system (Harvey et al., 2005). Many enhancements have been done recently, in finding either optimal robotic morphologies (Corucci et al., 2016) and adaptable robotic brains (Floreano et al., 2008). Hence, exploiting the interplay *robot-environment*, the evolutionary approach represents a model-free method to discover optimal locomotion patterns based on the interaction robot-terrain.

In this work, we present a new bio-inspired and model-free control architecture for quadruped robotic locomotion which takes advantages from the collaboration of evolution and adaptation. The evolutionary approach part for optimizing the Central Pattern Generator model on a simulated robot has already been investigated and tested (Urbain et al., 2018), while the cerebellar-like adaptive controller has been proven to be effective on both control of voluntary movements, such as control of a robotic arm (Tolu et al., 2012, 2013), and control of reflexes, such as in gaze stabilization tasks (Vannucci et al., 2016, 2017).

In comparison to the previous research works, where the evolutionary scenario is applied on the CPG parameters of the quadruped robot Tigrillo (Urbain et al., 2018), we proposed a comparative research proving the advantages of performing the evolution on an adaptive quadruped system *body + brain*. In the controller, the adaptive part is a cerebellar-inspired circuit (Tolu et al., 2012), which presents a modular structure for the quadruped locomotion task case. Further, for the first time, the paper shows the benefits of using the Cerebellar-inspired layer, already proposed by Ojeda et al. (2017), for robotic locomotion task.

To conclude and extend the result to a more general perspective, it is analyzed a comparison to the case where the evolution is performed just on the *body*, while the adaptive control part is included after the definition of the locomotion patterns, so after the findings of the locomotion trajectories by the evolutionary algorithm.

A comparison of the locomotion stability of the two bio-inspired controllers is then performed under different experimental constraints, to assess the generalizability of the results. These final experiments are very important because of the difficulty to transfer results found in simulation to the real world due to differences in sensing, actuation, and in the dynamic interactions between robot and environment. This phenomenon is called *reality gap* (Lipson and Pollack, 2000) and it is even more evident in adaptive approaches, where the control system is gradually designed and tuned through the repeated interactions between the agent and the surrounding scenario. Robots might evolve to match the specificities of the simulation, which differ from the real-world constraints. To prevent this problem, many approaches can be possible, such as adding independent noise to the values of the sensors or changing the robot dynamic model and its interaction with the environment (Nolfi et al., 2000; Vandesompele et al., 2019). In comparison to the classical approach where this simulation variability is added during the evolutionary optimization, in this research, the possibility of overcoming the reality gap and the transferability of the approach is demonstrated afterwards. Furthermore, to test the robustness of the proposed control architecture in the interaction with the environment, the static contact friction with the ground is changed during the test experiments. Usually, adaptive closed-loop CPG are exploited to counteract the changes in the environment (Kousuke et al., 2007; Ryu et al., 2010) while, in this research work, the learning and the adaptation of a cerebellar-inspired control module (Tolu et al., 2012) are applied instead to face the dynamically changing interaction with the external world.

The paper is structured as follows: in section 2 we describe the architecture of the controller, the evolutionary process employed and the implementation details; in section 3 we show the results of the evolutionary procedure and of the subsequent tests that have been performed; finally, in section 4 we discuss the obtained results and we draw the conclusions on the advantages of combining evolutionary processes and adaptive control.

## 2. Materials and Methods

In this work, a bio-inspired control architecture is implemented for the quadruped configuration of Fable robot (Pacheco et al., 2014), simulated on the Neurorobotics Platform (Falotico et al., 2017).

**Figure 1:**
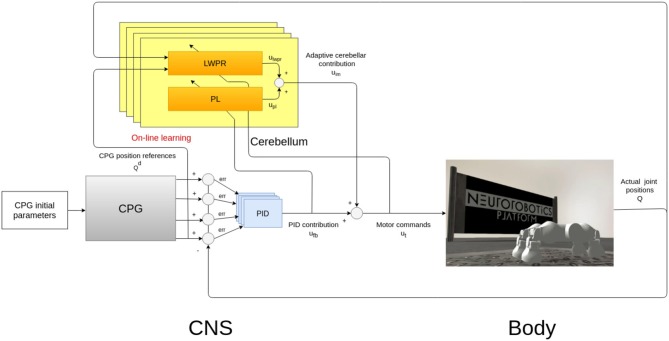
Bio-inspired control system design and implementation. The main modules of the architectures are a CPG-inspired trajectory planner, whose characteristic parameters have been chosen by a Covariance Matrix Adaptation Evolutionary Strategy (CMA-ES) (Hansen, 2006) approach and a Proportional “Integral” Derivative (PID) feedback controller which can cooperate with a cerebellar-inspired adaptive controller (Ojeda et al., 2017).

Figure 1 shows the system which consists of two parts: the controller, which is a simplified model of the CNS, comprising the CPG and the cerebellar circuit, and a simulated model of a quadruped robot, the Fable robot (Pacheco et al., 2014).

**Figure 2:**
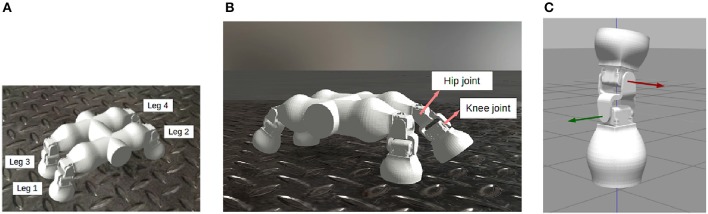
The Fable Robot in the Neurorobotics Platform (NRP). The robot has 4 legs **(A)** and 2 revolute joints per leg **(B)** which rotate around 2 perpendicular axes **(C)** (Pacheco et al., 2014; Falotico et al., 2017).

The robot has two degrees of freedom (DoF) for each leg (Figure 2A), but only one is actuated (the hip joint), while keeping the other fixed (Figure 2B) in order to reduce the number of parameters and simplifying the evolutionary process. This simplification does not pose a problem, as locomotion patterns can still be achieved by only using the hip joints.

### 2.1. Central Pattern Generator (CPG)

In quadruped biological systems, simple locomotion can be generated as a low-level brain function, in the spinal cord, in the form of CPG. The term *central* indicates that there is no need for peripheral sensory feedback to generate the rhythms. From a control point of view, the CPG has also very interesting properties such as distributed control and modulation of locomotion by simple high-level commands (Ijspeert, 2008).

In our system, this biological neural function is mathematically modeled as a network of coupled non-linear oscillators and they are represented as the gray box in Figure 1 (Gay et al., 2013). These oscillators are then used to plan the angular excursion in time of the hip joints of a quadruped robot (Figure 2). The benefits of using these oscillators lie in the fact that they are controlled by a low number of parameters that specifically affect certain aspects of the locomotion pattern. For instance, one of the most relevant parameters is the duty cycle (*d* in Equation 4) which controls the shape of a skewed sine wave modulating the protraction-retraction of the hip joint of the robot as shown in the systems of equations 1-4.

The CPG module is the main block involved in the evolutionary procedure (Sect. 2.3) and it is implemented in open-loop in the control architecture.

The initial parameters and the boundaries of the oscillators (Table 1), employed as a CPG, are selected to be a general starting point for the optimization algorithm. In defining the variables of the CPG oscillators, a difference between the front and hind legs is made to better characterize the morphology of the robot and to follow the default specifications of the work by Gay et al. (2013). These variables are the deterministic specifications which induce a certain type of locomotion for the Fable robot. Indeed, the locomotion patterns represent the *phenotype* for the evolutionary process, which means that they are the observable characteristics resulting from the interaction of the genotype of the robot with the environment. Equally, the CPG parameters (Table 1) represent the *genotype* which is evolved and mutated through multiple generations, whose expression are de facto the locomotion patterns (phenotype). In fact, to not steer the evolution toward a limited area in the space of the possible genetic outcomes, the generalizability and unbiasedness of the starting values of the genotype are fundamental.

**Table 1:** Distinctive parameters of the coupled oscillators which define the four joint trajectories for the robot.

**Parameters**	**Initial values**	**Boundaries**		**Results**	
		**Min**	**Max**	***adapt-after-evo***	***adapt-in-evo***
**CPG EVOLVED PARAMETERS**					
Front legs amplitude (μ)	1.58	0.5	1.56	1.04	1.46
Hind legs amplitude (μ)	0.88	0.5	1.56	0.69	0.71
Frequency (ω)	5	1	10	4.9	8.57
Phase shift leg 1-2 (ϕ)	0.001	0	6	1.19	0.38
Phase shift leg 2-3 (ϕ)	1.14	0	6	5.9	3.42
Phase shift leg 3-4 (ϕ)	4.35	0	6	1.4	3.32
Duty cycle leg 1 (*d*)	0.12	0	0.9	0.88	0.29
Duty cycle leg 2 (*d*)	0.75	0	0.9	0.57	0.73
Duty cycle leg 3 (*d*)	0.40	0	0.9	0.84	0.28
Duty cycle leg 4 (*d*)	0.85	0	0.9	0.9	0.69
Offset left front leg (*o*)	−20.8	−60	60	−12.73	22.3
Offset right front leg (*o*)	18.53	−60	60	36.58	−10.9
Offset left hind leg (*o*)	−17.96	−60	60	57.24	52.47
Offset right hind leg (*o*)	52.72	−60	60	8.64	26.79

*These parameters define the four outputs of the Central Pattern Generator described in Gay et al. (2013) and their values are evolved during the CMA-ES search for the optimal solutions (Hansen, 2006) either in the adapt-after-evo and in the adapt-in-evo*.

The selected parameters are listed in Table 1, where their initial values, boundaries and final optimal results are presented.

Here below, the equations of the unit oscillators model for the *i* − *th* robotic hip, with ϕ_2π_ = ϕ_*i*_(mod 2π):

(1) $$\begin{array}{r} {ṙ}_{i}=\gamma \left(\mu_{i}-r_{i}^{2}\right)r_{i} \end{array}$$

(2) $$\begin{array}{r} {\overset{∙}{\phi }}_{i}=\omega_{i}+\overset{4}{\underset{j=1}{\sum }}w_{ij}\sin\left(\phi_{j}-\phi_{i}-\psi_{ij}\right) \end{array}$$

(3) $$\begin{array}{r} \theta_{i}=r_{i}\cos\left(\phi_{L_{i}}\right)+o_{i} \end{array}$$

(4) $$\phi_{L_{i}}\;=\;\{\begin{array}{cc} \frac{\phi_{2\pi }}{2d_{i}} & if\;\phi_{2\pi \;<\;2\pi d_{i}}\\ \frac{\phi_{2\pi \;+\;2\pi \left(1\;-\;2d\right)}}{2\left(1-d_{i}\right)} & otherwise \end{array}$$

*r* is the radius of the hip oscillator, μ is its hip target amplitude, ω its frequency, ϕ its phase, *o* its offset and θ its output angular excursion in radians. γ is a positive gain defining the speed of convergence of the radius to the target amplitudes μ. *d* is the virtual duty factor since the actual duty factor depending on the robot dynamics and on parameters of the gait. The four hips of the robot are also phase-coupled to synchronize them, to achieve different gaits. More in details, the coupling between hip oscillators *i* and *j* is obtained by adding the term *w*_*ij*_*sin*(ϕ_*j*_ − ϕ_*i*_ − ψ_*ij*_) in Equation (2), where ψ_*ij*_ is the desired phase difference between the oscillators controlling hips *i* and *j* and *w*_*ij*_ is a positive gain. Eventually, ϕ_*L*_ (Equation 4) is a filter applied on the phase ϕ and cos(ϕ_*L*_) is used to compute the output angle θ of the hip oscillator.

The described CPG oscillator acts as a trajectory planner in the control architecture since coordinates the robotic motion, defining the locomotion characteristics. In quadrupeds, the neural signal which descends from the spinal cord along the motoneurons regulates the contraction of the peripheral muscle fibers (Takakusaki, 2013). To obtain a consistent motor control signal, the final signals sent to the robotic legs are joint efforts. In the case of the Fable robot, these efforts are motor torques, computed by a PID feedback controller, after the CPG planning (Figure 1).

### 2.2. Bio-inspired Adaptive Controller

The proposed bio-inspired controller (in light blue and yellow in Figure 1) mimics one of the cerebellar roles in locomotion: the computation of the feedback-error-learning model. The body, or a part of the body as a leg, is a physical entity whose movements are controlled by the CNS. The controlled entity can be considered as a cascade of transformations between motor command (e.g., muscle activations in the biological case and joint torques in the robotic one) and links motion (e.g., joint angular position). This cascade of transformations defines the system dynamics. The neural description, which models the transformation from the desired movement trajectory to the motor commands needed to obtain it, is called the *inverse model*. This concept explains that if the inverse model is accurate, it can be used as a feedforward controller, making the actual trajectory be reasonably comparable to its reference (Wolpert et al., 1998).

The proposed controller is then composed by a feedback part and a bio-inspired part (Tolu et al., 2012). The feedback part element is a PID controller (in light blue in Figure 1), often used in engineering for torque control, while the bio-inspired one is a simplified model of a cerebellar circuit (in yellow in Figure 1).

The cerebellar-inspired model has the role of computing a corrective torque contribution based on the inverse model of the system. As in the biological cerebellum, a specific circuit is dedicated to the inverse model of each one of the legs, but still merging information concerning the global body/robot state. Each circuit works as a Unit Learning Machine (ULM) which encodes the internal model of a body part to more precisely perform more precise motion control (Ito, 2008).

**Figure 3:**
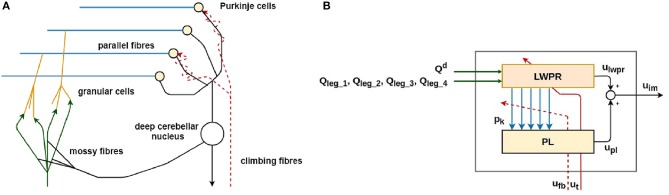
The simplified biological model of the cerebellar microcircuit **(A)** and its functional and computational implementation **(B)** (Ojeda et al., 2017). The implementation of the main parts of the biological cerebellar model **(A)** is represented in the same color in the corresponding control block **(B)**.

In Figure 3, the simplified model of one of the four biological cerebellar microcircuits and its mathematical implementation is shown.

The main functional biological sub-parts in the cerebellar microcircuit are:

- the **Mossy fibers (MF)**: they transfer the sensory inputs to the cerebellum (green in Figure 3);
- the **Granular cells (GC)**: they expand the sensory information from the mossy fiber to abstract the inverse model of the body movement corresponding to the specific body part (orange in Figure 3);
- the **Parallel fibers (PF)**: they transmit the information from the granular cells to the Purkinje cells. This layer is shared among all the cerebellar microcircuits and represents where the information is shared among the four cerebellar modules (light blue in Figure 3);
- the **Purkinje cells (PC)**: they modulate the input from the granular cells, which is carrying information about the actual state of the robot. The modulation is performed thanks to teaching information coming from the inferior olives through the climbing fiber (yellow in Figure 3);
- the **Climbing fibers**: they carry the teaching signal to the Purkinje cells to modulate their activity (red dashed line in Figure 3);
- the **Deep nuclear cell (DCN)**: it gathers and integrates inputs from the information elaborated by the Purkinje and Granular cells. It generates the final cerebellar output (white in Figure 3).

The cerebellar inspired control module contains a total of 4 ULMs, one for each leg (Figure 1). Each ULM is considered as a single cerebellar microcircuit and the communication and synchronization through the different circuits are provided by the PFs layer and encoded as the information *p*_*k*_ in the Equation (3). *p*_*k*_ is also transferred between two sub-modules of the learning machine (in light blue in Figures 3A,B). Each microcircuit consists of 3 modules: a module for the *cortical layer* of the cerebellum (in orange in Figure 3), a module for its *molecular layer*, mainly constituted by the *Purkinje Cells Layer (PL)* (in yellow in Figure 3), and eventually, a model of the *Cerebellar Nuclei (DCN)* (the white circle in Figure 3B). All modules contribute to computing the final corrective command which constitutes the inverse model effort contribution *u*_*im*_ to the robot.

More in detail, the *cortical layer* module is implemented through the Locally Weighted Projection Regression (LWPR) algorithm. The LWPR is an algorithm for incremental nonlinear function approximation in high-dimensional spaces with redundant and irrelevant input dimensions (Vijayakumar and Schaal, 2000). This machine learning technique is computationally efficient and numerically robust thanks to its regression algorithm; it creates and combines *N* linear local models which perform the regression analysis in selected directions of the input space, taking inspiration from the partial least squares regression. The main advantages of using the described learning algorithm are listed in the following:

- it optimizes the role of the GC in the cerebellum, which exploit their particular plasticity to learn the dynamic model of the body for motor control (orange in Figure 3);
- it acts as a radial basis function filter which implies the processing of the sensory information input from the MF to the DCN (*p*_k_ in Equation 7 and in black in Figure 3);
- it allows rapid learning based on incremental training which perfectly fit in the specification of the designed system which should be able to perform online learning, based on the dynamical environmental constraints;
- its learning is extremely fast and accurate since the weights of each kernel is based only on local information and its computational complexity is linear for each input information.

Each LWPR model is fed with the sensory inputs which are the reference position for the specific leg hip joint (*Q*^*d*^) and the actual positions (*Q*_*leg*_*y*__ for *y* in *ULMs*) of all the 4 controlled joints. Then, the algorithm performs an optimal function approximation and divides the sensorimotor input space into a set of receptive fields (RFs), which represent the neurons of the cerebellar GCs layer. The RFs geometry is described by Equation (5), which describes a Gaussian weighting kernel. For each multidimensional input data point *x*_*i*_, a RF activation *p*_*k*_ is computed, based on its distance to the center of the Gaussian kernel *C*_*k*_.

(5) $$\begin{array}{r} p_{k}\left(x_{i}\right)=e^{-\frac{1}{2}\left({\left(x_{i}-c_{k}\right)}^{T}\cdot D_{k}\left(x_{i}-c_{k}\right)\right)} \end{array}$$

Basically, each RF activation *p*_*k*_ is an indicator of how often an input happens to be in the validity region of each RF linear model. The validity region is defined by a positive definite distance matrix *D*_*k*_. The distance matrix is updated at each iteration according to a stochastic leave-one-out cross-validation technique to allow stable on-line learning. At each iteration, the LWPR weights *p*_*k*_ are sent to the cerebellar *molecular layer* model and once that the optimal centers and widths are found for each RF, the accuracy and the learning speed increase. Equation (3) has been proved to lead to a sparse code of the input data *x*_*i*_ and this facilitates the persistence of remaining sites of plasticity for the incremental learning process, as in the biological cerebellar circuit (Dean et al., 2010).

The output of the *k*_*th*_ RF is shown in Equation (4), where *w*_*k*_ is the weight vector of the RF and ϵ_*k*_ is the bias.

(6) $$\begin{array}{r} y_{k}\left(x_{i}\right)=w_{k}x_{i}+\epsilon_{k} \end{array}$$

Moreover, the LWPR acts as a radial basis function filter which elaborates the sensory information and returns it as *u*_*lqpr*_ (Equation 7), that is the contribution from the cortical layer of the cerebellar microcircuit model. This contribution is modeled as a weighted linear combination of the kernels outputs *y*_*k*_(*x*_*i*_).

(7) $$\begin{array}{r} u_{lwpr}\left(x_{i}\right)=\frac{\sum_{k=1}^{N}p_{k}\left(x_{i}\right)y_{k}\left(x_{i}\right)}{\sum_{k=1}^{N}p_{k}\left(x_{i}\right)} \end{array}$$

*p*_*k*_ (Equation 3) also represents the contribution which is transmitted through the parallel fiber to the Purkinje Layer (PL). The parallel fibers gather all the information from the different GCs kernels. This information is multiplied by a set of weight *r*_*k*_ and thus, we obtain *u*_*pl*_, the Purkinje Cell Layer (PL) output (Equation 6).

(8) $$\begin{array}{r} u_{pl}\left(x_{i}\right)=\underset{k}{\sum }r_{k}p_{k}\left(x_{i}\right) \end{array}$$

The learning rule used for updating the weights in the Purkinje Cells Layer is explained in Equation (7), where the update gain δ_*r*_*k*__ is computed. β is a small learning rate (usually 0.07) and *u*_*fb*_(*x*_*i*_) is the motor command from the feedback part of the controller, used as teaching signal.

(9) $$\begin{array}{r} \delta_{r_{k}}=\beta u_{fb}\left(x_{i}\right)p_{k}\left(x_{i}\right) \end{array}$$

Taking inspiration from the biological cerebellar micro-structure, the final output of the entire cerebellar circuit is the neural command coming from the Deep Cerebellar Nucleus (DCN) or Deep Nuclear Cell which represents the inverse model corrective torque *u*_*im*_ (Equation 8).

At each simulation iteration, the total effort command *u*_*t*_ to be sent to the robot is computed as in the Equation (8).

(10) $$\begin{array}{r} u_{t}\left(x_{i}\right)=u_{fb}\left(x_{i}\right)+u_{im}\left(x_{i}\right)=u_{fb}\left(x_{i}\right)+u_{lwpr}\left(x_{i}\right)+u_{pl}\left(x_{i}\right) \end{array}$$

### 2.3. Evolutionary Algorithm

In evolutionary robotics, the desired robotic behaviors emerge automatically through evolution due to the optimization and interactions between the robot and its surrounding environment. As a specification for the evolutionary procedure, a fitness function, which measures the ability of a robotic individual to perform the desired task, is defined based on this optimization procedure, the algorithm identifies the optimal robotic configuration (Pratihar, 2003).

In this research, an evolutionary algorithm to optimize the initial parameters of the CPG is applied using a *covariance matrix adaptation evolutionary strategy (CMA-ES)* (Hansen, 2006). It is a stochastic optimization algorithm which, compared to other evolutionary procedures, has the advantage of converging rapidly in a landscape with several local minima and requires few initialization parameters (Hansen, 2006). In an iterative fashion, the algorithm changes the initial CPG parameters (Table 1) and simulates the resulting locomotion patterns on the simulated robotic platform for 2 min. At the end of the simulation, a fitness function computes a score to give to the different individuals, based on the distance each robot has covered during the locomotion simulation. The initial parameters for the CMA-ES are implemented as described by Hansen (2006).

### 2.4. Experimental Design

**Figure 4:**
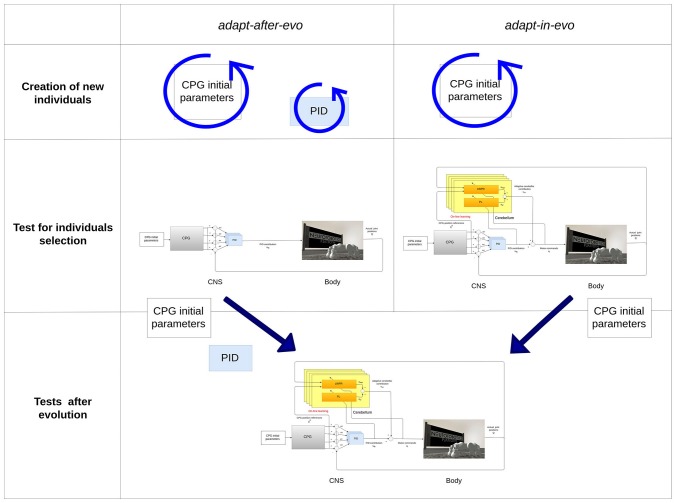
Description of the two systems *adapt-after-evo* and *adapt-in-evo*. On top, during the evolution, the *adapt-after-evo* evolves the initial parameters of the CPG and the PID gains and during the experiments, the bio-inspired module is plug in the architecture. On the bottom, the *adapt-in-evo* architecture keeps the PID gains fixed to the initial values of the same values for the *adapt-after-evo*.

To assess the advantages of exploiting adaptability in employing evolution strategies for robotic locomotion tasks, two different configurations of the system are evolved (Figure 4):

- **adapt-after-evo:** Co-evolution of the CPG parameters and PID gains (Tables 1, 2)
  – *genotype:* CPG parameters + PID gains
  – *phenotype:* locomotion patterns
- **adapt-in-evo:** Evolution of the CPG parameters + learning phase of the cerebellar circuit (fixed PID gains, Tables 1, 2)
  – *genotype:* CPG parameters
  – *phenotype:* locomotion patterns + RFs in the cerebellar circuit

**Table 2:** PID gains and hyper-parameters of the Cerebellum-inspired controller.

**PID parameters**	***Adapt-after-evo***			***Adapt-in-evo***
	**Boundaries**		**Result**	**values (fixed)**
	**Min**	**Max**		
Kp	0.5	1	0.86	0.81
Ki	0.001	0.009	0.005	0.005
Kd	0.02	0.06	0.022	0.040

*In the adapt-after-evo, the K_p_, K_i_, and K_d_ are evolved in the CMA-ES, as the CPG parameters in Table 1, while in the adapt-in-evo they are fixed as the initial values of the adapt-after-evo. Concerning the remaining four parameters, they are specifications for the learning modules of the architecture and for that, they are used just in the adapt-in-evo*.

The PID gains are part of the evolved parameters in the *adapt-after-evo* in order to have a fair comparative study of the performance of the two systems. The classic controller (the *adapt-after-evo*) should be also optimized by the evolutionary exploration. Their initial conditions and the boundaries for the CPG parameters are the same, as in Table 1.

As a starting point for the evolution, the PID gains are the same for both robotic configurations: *adapt-after-evo* and *adapt-in-evo*. In the *adapt-after-evo* configuration, the PID gains are part of the evolutionary process and their boundaries are defined according to empirical evaluations on the stability of the system, while in the *adapt-in-evo* system configuration when the cerebellar circuit is plugged in the system, they are fixed (Figure 4, Table 2).

Concerning the specification of the cerebellar circuit, an experimental tuning has been performed on four of the most significant hyper-parameters of the LWPR algorithm (Vijayakumar and Schaal, 2000) (*init_D, init_*α, *w_gen* and *add_threshold* in Table 2), to obtain a stable and corrective system behavior for the frequency range of the locomotion trajectories (ω in Table 1), used as starting point of the evolutionary algorithm. This is an important constraint for the experiments because the response of the system needs to be stable for all the possible solutions found by the evolutionary algorithm. Ensuring stability in the system allows inspecting an unbiased comparison even if the adaptive part of the controller is included afterwards.

The first two hyper-parameters considered (*init_D* and *init_*α) are related to the creation of new Receptive Fields, while the last two (*w_gen* and *add_threshold*) directly influence the local regression algorithm. All the hyper-parameters are the same for the 4 Unit Learning Machines and they are described as follows:

- **init_D** = 0.7, it represents the initial distance metric which is assigned to each new created Receptive Fields (RFs);
- **w_gen** = 0.6, it is critical for the creation of new RFs. If no local model shows an activation greater than this value, a new RF is generated;
- **init_α** = 500, it is the initialization value for the learning rate in the gradient descent algorithm which minimizes the error in the different regressions of the input space;
- **add_threshold** = 0.95, it operates as a threshold value to stand when a new regression direction should be added to the algorithm. If the ratio between the mean squared error of the current regression dimension and the same mean squared error, at the previous time iteration, is lower than this value, thus, a new regression direction can be exploited in the robot modeling process.

All the simulations were run on the Neurorobotics Platform and implemented through its utilities, which has been shown capable of implementing robotic control loops (Vannucci et al., 2015). The controller was implemented using a domain-specific language that eases the development of robotic controllers, and that is part of the Neurorobotics Platform simulation engine (Hinkel et al., 2017). Another tool, called *Virtual Coach* and also included in the platform and employed to implement the evolutionary algorithm. It was used because capable of launching batch simulations with different parameters and gathering and storing results from these.

## 3. Experimental Results

In both evolutionary configurations, each of the 16 generations consists of 10 individuals. Every simulation lasted for 2 min, which is enough time for the LWPR to converge. After the simulation, the fitness function has been computed.

In Table 1, the resultant characteristic parameters of the final CPG configurations for the best individuals in the *adapt-after-evo* and *adapt-in-evo* configuration, are shown.

In Table 2, for the*adapt-after-evo*, the PID gains are part of the genotype and their initial conditions represent the same fixed controller parameters used for the *adapt-in-evo*. Thus, in the*adapt-after-evo* case, the PID gains are changed by the evolutionary process, within the experimentally found boundary conditions for the starting locomotion robotic patterns to be stable and tolerable. Differently, the *adapt-in-evo* profits from the contribution of the cerebellar-inspired controller (Figure 3B), whose hyper-parameters (*init_D, init_*α, *w_gen* and *add_threshold*) are set as shown in Table 2 and explained in section 2.4.

After the evolutionary process, experiments that compare the behavior of the two systems have been performed. To perform this comparison, the same cerebellar circuit, that was used in the *adapt-in-evo*, was plug in the *adapt-after-evo*. Thus, both systems are now adaptive thank to the contribution of the cerebellar control module and it is possible to test and compare the benefits of control adaptability *during* or *after* the optimization of the planning of the locomotion trajectories. The two resultant control architectures are then representative for:

- control adaptability during the evolutionary optimization of the CPG locomotion patterns (*adapt-in-evo*)
- control adaptability after the evolutionary optimization of the CPG locomotion patterns (*adapt-after-evo*)

While the individual representative for the *adapt-in-evo* architecture can safely be chosen as the winner of the evolutionary algorithm, the effect of adding the adaptive component to create the *adapt-after-evo* cannot be easily predicted. Thus, in order to better choose the individual for the *adapt-after-evo* architecture, the cerebellar circuit was added to the best three individuals resulting from the evolutionary process. After evaluating again, the fitness with the adaptive component, the one individual with better performances was chosen as the representative one.

In general, to provide a fair comparison between the two systems, the distance is computed only after the cerebellar algorithm has converged, as in the initial phase, where learning occurs, we can observe some instability. After this initial phase, that lasts for around 20 s, we can notice no significant improvements in the position error on the joint trajectories, which could indicate that most of the learning has been done. This can also be observed by looking at the number of receptive fields created by the LWPR algorithm, that is not increasing anymore. Therefore, to avoid having the learning phase affecting the computation of the distance covered by the robot, a time window of 20 s is considered, from 30 to 50 s, during which the distance covered by the robot is recorded and compared between the two different cases (*adapt-after-evo* and *adapt-in-evo*).

### 3.1. Base Comparison

After simulating the best *adapt-after-evo* and *adapt-in-evo* individuals 10 times for 1 min, the results show that the winner robot walks for 1.72 m on average with the *adapt-in-evo* controller while it walks for 1.48 m with the *adapt-after-evo*. The respective standard deviations are 0.2 m for the *adapt-in-evo* controller and 0.11 m for the *adapt-after-evo*. This shows that, in the task space, there are benefits in using the adaptive controller during the search for the best locomotion patterns, rather than connecting it to the control architecture afterwards. The superiority of the *adapt-in-evo* approach is raised also by the fact that PID gains are no evolved and they keep the values, presented in Table 2, while the same gains are optimized in the *adapt-after-evo* approach.

**Figure 5:**
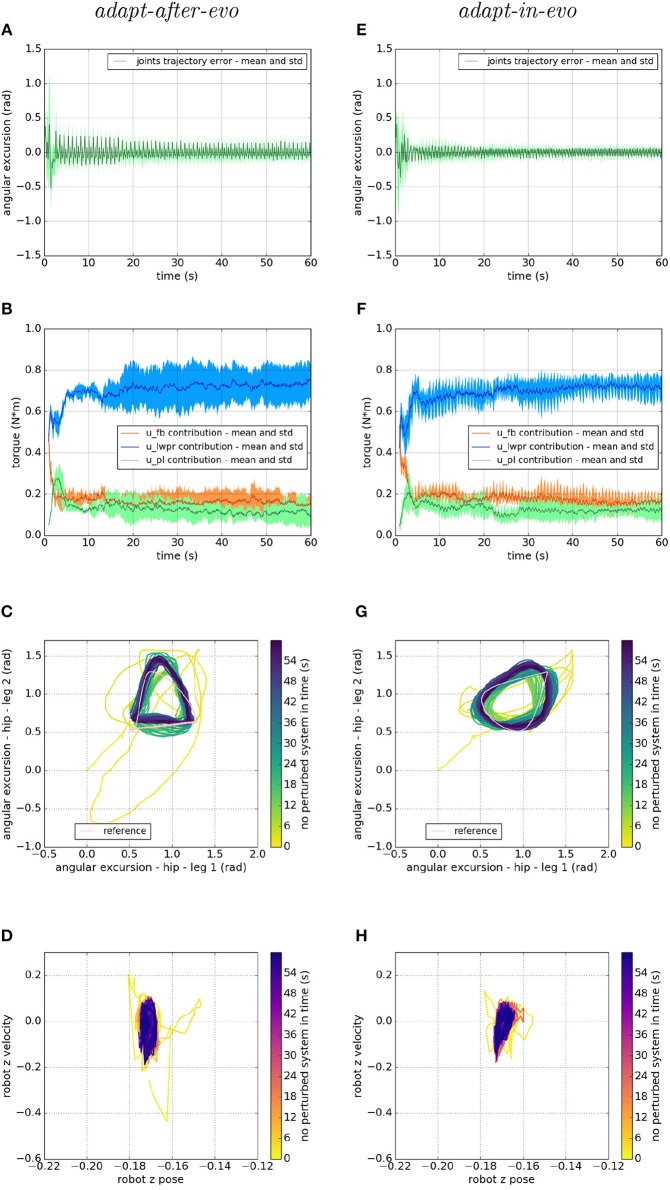
Locomotion performance and characterization of the two systems; on the left the *adapt-after-evo* system and, on the right, the *adapt-in-evo* one. **(A,E)** Represent the mean and the standard deviation of the position error of the four legs joints and **(B,F)** instead describe the mean and the standard deviation of the contribution ratio of the different modules of the control architecture. **(C,G)** Describe the periodic behavior relation between the actual joint trajectories of leg 1 and leg 2 compared to their reference values, in pink (among the other pairs of legs, the relation is periodic in a comparable way). Eventually, **(D,H)** represent the dynamics of the CoM of the robot, on the vertical axis to the ground. By plotting the CoM velocity against its position on the vertical axis, we can extract relevant information about the stability of the locomotion.

Regarding the behavior of the two systems in the joint space, we analyze the differences in their performances as shown in Figure 5. On the left column, the *adapt-after-evo*-related plots are shown and on the right column, the plots related to the *adapt-in-evo*-system are presented.

Figures 5A,E represent the mean and the standard deviation of the position error of all the robotic legs. In both pictures, after an overshoot at the beginning of the simulation, which represents the transient where the cerebellar controller is calibrating its corrective contribution, the error decreases along with the simulation. Comparing the two plots, it is appreciable that in the *adapt-in-evo* trial (e) the error in following the reference positions is almost half compared to the other case *adapt-after-evo* (a). Their Root Mean Square Error (RMSE) are, respectively, 0.035 radians and 0.056 radians.

Then, in Figures 5B,F, the mean and the standard deviation of the ratio of the contributions of the different parts of the bio-inspired cerebellar controller are highlighted. It is evident that, in both cases, the contribution of the LWPR, whose teaching signal is the global motor command to the robot *u*_t_, becomes predominant compared to the feedback controller contribution (PID). Furthermore, the PL contribution, whose teaching signal is the feedback controller *u*_fb_, follows the trend of the output of the PID controller, which decreases along with the simulation, meaning that the final motor commands to the robot are mostly relying on the *u*_*im*_ output.

On the third line, Figures 5C,G stress the periodic and stable locomotion which characterizes the system after the first seconds of simulation. In the Figures 5C,F, just the cyclic behavior of two robotic legs (leg 1, one of the front legs, and leg 2, one of the hind legs) has been reported. The remaining two legs present comparable performances. It is appreciable from Figures 5C,G that the relation among the angular excursions of the two legs becomes more periodic along with the simulation time and closer to the pink limit cycle, shown to mark the reference trajectories of leg 1 and leg 2.

Ultimately, at the level of the task space, a dynamic analysis of the robotic locomotion is exhibited in Figures 5D,H when the robot vertical position is plotted against its vertical speed. In these images (Figures 5D,H), the dynamics of the system become more defined and constrained over time. It is relevant to point out that, in the *adapt-in-evo* case (Figure 5H) the winner locomotion patterns grant more robust locomotion, which is represented by a more confined stability region in the phase space with respect to the *adapt-after-evo* system (Figure 5D).

### 3.2. Statistical Analysis on Different Experimental Conditions

After discussing the results concerning the advantages of using control adaptability during the optimization of the locomotion trajectories (*adapt-in-evo*) rather than employing it afterwards (*adapt-after-evo*), we investigated on the effects of altering the experimental conditions with respect to the simulation circumstances where the locomotion patterns have been found. These experiments are also useful for testing the system in more realistic scenarios, which goes toward overcoming the reality gap. The adaptation to the changes in the experimental scenario is possible since the weights of the LWPR are never locked to certain values, but they are always updating based on the experimental circumstances.

The changes in the experimental constraints have been applied in the following order:

- variability in the robotic dynamics;
- variability in the interaction with the environment.

First, to verify the abstraction potential of the previous results, a population of 15 slightly different Fable robots is generated. After checking the consistency of the simulation in a certain range of variation of the robotic model dynamic parameters, we decided to generate 15 robots with the following features:

- additive white Gaussian noise (AWGN) fed in the encoder of the motors and randomly selected from a uniform distribution in the range of [0–10] % of the motor signal;
- damping coefficient, randomly taken from a uniform distribution in the range of [0.08–0.25] $\frac{Ns}{m}$, to define the dynamic model of all the hip joints of the robot.

Thus, the resulting 15 Fable robots have different dynamic characteristics and noisy signals injected in their motors' encoder. These modifications model the variability in the robotic population.

Subsequently, other experimental constraints have been modified. They represent the variability in the interaction robot-environment. Thus, to modulate this aspect of the simulation, the static friction coefficient is altered in the *x*−*direction* of the world reference frame. The default value of the simulator for this parameter is 1, meaning maximum static friction between robot and ground and we decided to affect the experiments by giving three different levels: 0.3, 0.5, 0.95 of static friction coefficient to the interaction robot-ground. Lower coefficients imply greater disturbances to the system. To have consistent results, the previously generated robotic individuals are simulated ten times for 1 min in each of the 3 different friction conditions explained above.

**Figure 6:**
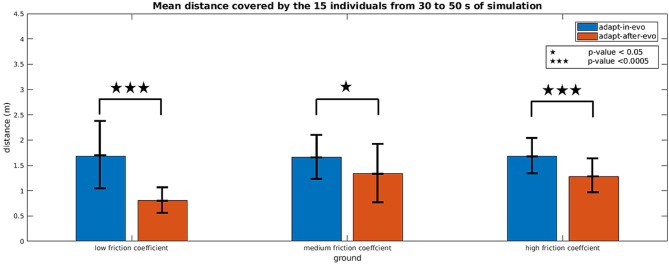
Histograms which summarize the mean and standard deviation of the distance covered by the 15 individuals with the two control strategies *adapt-in-evo* and *adapt-after-evo* in the three different levels of robot-ground friction. The *p*-values, regarding the statistical significance of the performance of the two system *adapt-in-evo* and *adapt-after-evo*, are also shown in the figure.

Figure 6 shows histograms with an error bar for the mean and standard deviation of the distance covered by all the combinations robot-terrain, simulated with the two different control architectures *adapt-after-evo* and *adapt-in-evo*, 10 times per individual.

A two-way repeated measures ANOVA (Potvin and Schutz, 2000) was run to determine the effect of the two systems (*adapt-in-evo*, and *adapt-after-evo*), i.e., factor *controller* over three different ground-robot interactions (low, medium and high friction), i.e., factor *ground* on the explanatory variable walked distance (*D*), expressed in meters. Data are mean ± standard deviation. Analysis of the studentized residuals showed that there was normality, as assessed by the Shapiro-Wilk test of normality (Razali and Wah, 2011) and no outliers, as assessed by no studentized residuals greater than ± 3 standard deviations. The assumption of sphericity was violated for the interaction term, as assessed by Mauchly's test of sphericity (*X*^2^(2) = 7.003, *p* = 0.03) (Gleser, 1966). There was a statistically significant interaction between *controller* and *ground* on *D, F*_(1.412, 19.767)_ = 4.288, *p* = 0.04, ϵ = 0.706 (Greenhouse-Geisser correction Abdi, 2010), partial ν^2^ = 0.234.

**Figure 7:**
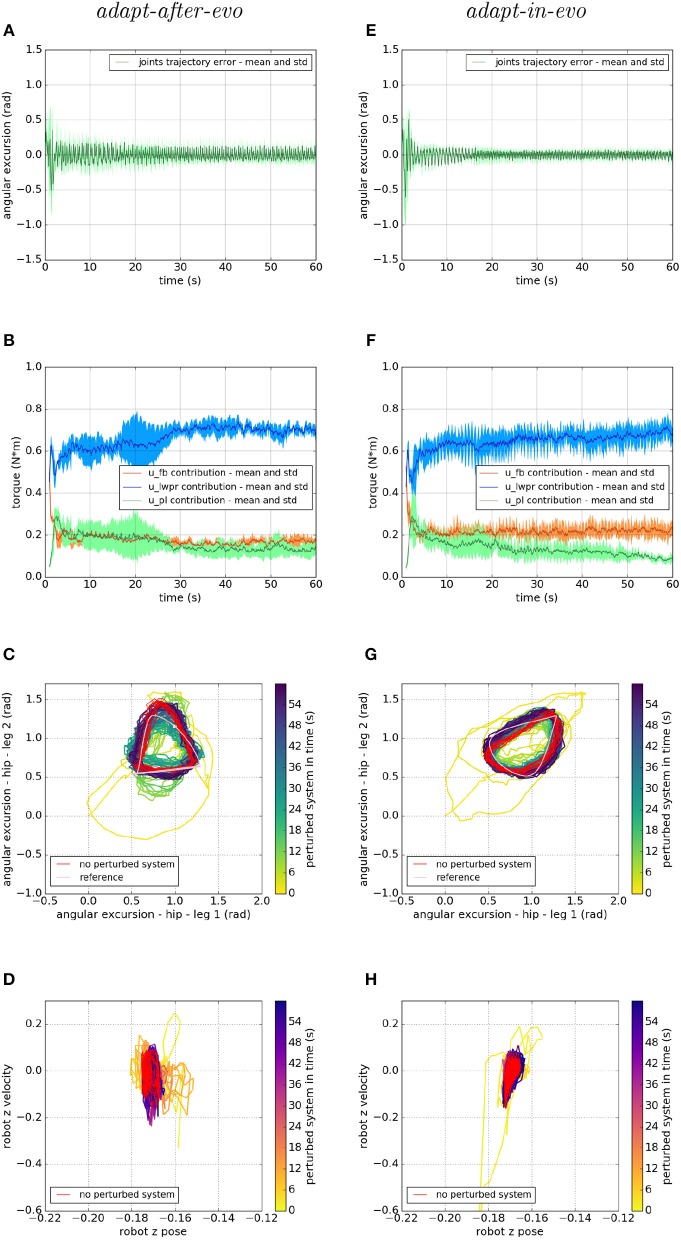
Locomotion performance and characterization of the two systems; on the left the *adapt-after-evo* system and, on the right, the *adapt-in-evo* one, with a friction coefficient of 0.95 between robot and terrain. **(A,E)** Represent the mean and the standard deviation of the position error of the four legs joints and **(B,F)** instead describe the mean and the standard deviation of the contribution ratio of the different modules of the control architecture. **(C,G)** Describe the periodic behavior relation between the actual joint trajectories of leg 1 and leg 2 compared to their reference values, in pink, and to the behavior of the no perturbed system, in red (among the other pairs of legs, the relation is periodic in a comparable way). Eventually, **(D,H)** represent the dynamics of the CoM of the robot, on the vertical axis to the ground, compared the same CoM dynamics when the system is not perturbed (in red).

**Figure 8:**
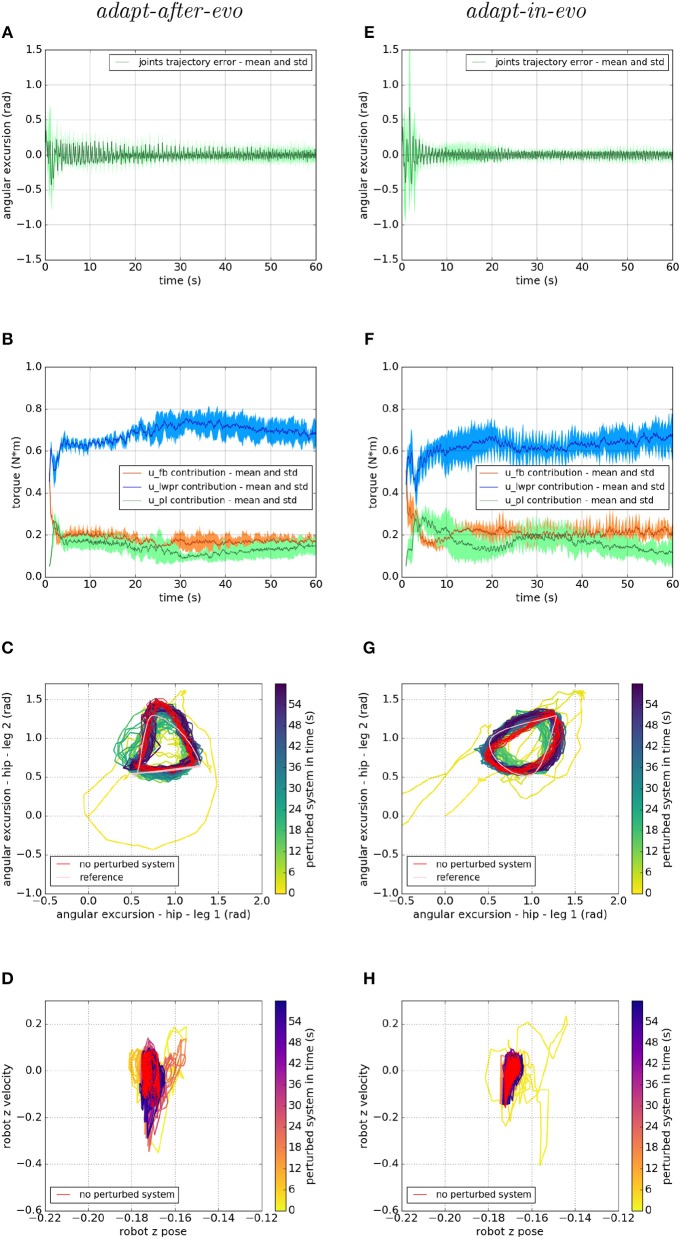
Locomotion performance and characterization of the two systems; on the left the *adapt-after-evo* system and, on the right, the *adapt-in-evo* one, with a friction coefficient of 0.5 between robot and terrain. **(A,E)** Represent the mean and the standard deviation of the position error of the four legs joints and **(B,F)** instead describe the mean and the standard deviation of the contribution ratio of the different modules of the control architecture. **(C,G)** Describe the periodic behavior relation between the actual joint trajectories of leg 1 and leg 2 compared to their reference values, in pink, and to the behavior of the no perturbed system, in red (among the other pairs of legs, the relation is periodic in a comparable way). Eventually, **(D,H)** represent the dynamics of the CoM of the robot, on the vertical axis to the ground, compared the same CoM dynamics when the system is not perturbed (in red).

**Figure 9:**
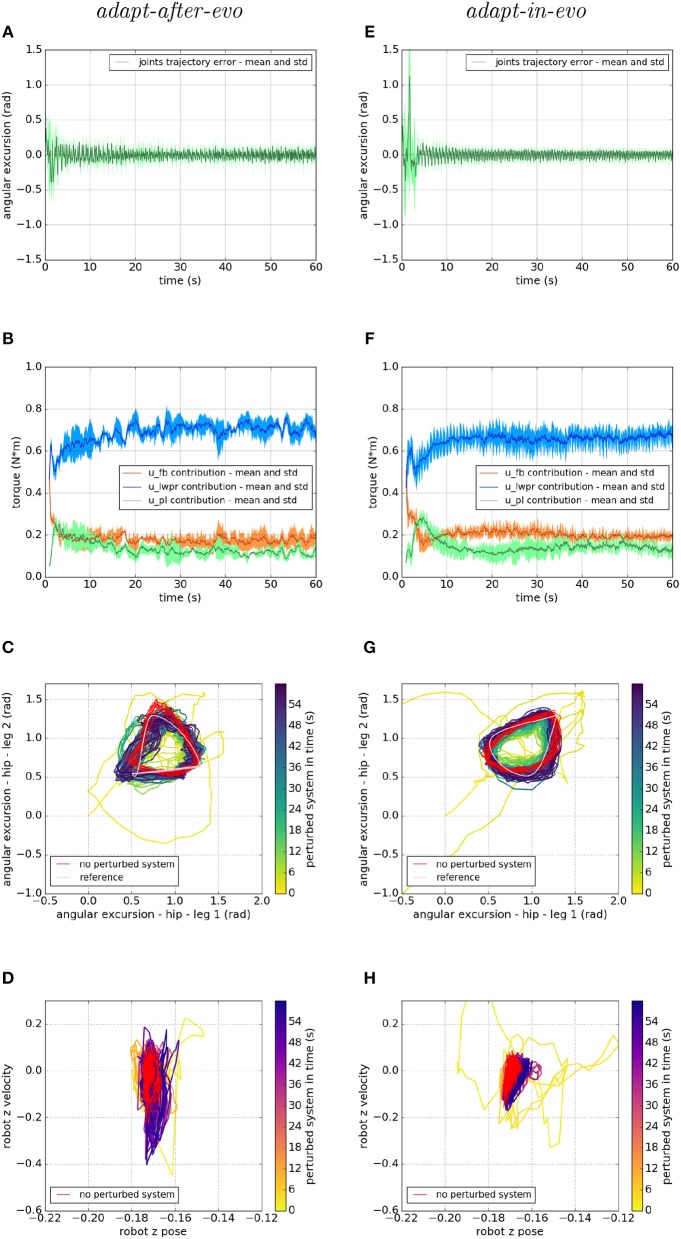
Locomotion performance and characterization of the two systems; on the left the *adapt-after-evo* system and, on the right, the *adapt-in-evo* one, with a friction coefficient of 0.3 between robot and terrain. **(A,E)** Represent the mean and the standard deviation of the position error of the four legs joints and **(B,F)** instead describe the mean and the standard deviation of the contribution ratio of the different modules of the control architecture. **(C,G)** Describe the periodic behavior relation between the actual joint trajectories of leg 1 and leg 2 compared to their reference values, in pink, and to the behavior of the no perturbed system, in red (among the other pairs of legs, the relation is periodic in a comparable way). Eventually, **(D,H)** represent the dynamics of the CoM of the robot, on the vertical axis to the ground, compared the same CoM dynamics when the system is not perturbed (in red).

Simple main effects were run for the factor *controller* (Figure 6). *D* of *adapt-in-evo* controller was always higher than that of *adapt-after-evo*:

- data for low-friction *ground* were (1.69 ± 0.71) m and (0.80 ± 0.27) m, respectively, *p*-value < 0.0005 (3 stars in Figure 6);
- data for medium-friction *ground*, (1.66 ± 0.45) m and (1.34 ± 0.55) m, respectively, *p*-value < 0.05 (1 star in Figure 6);
- data for high-friction *ground*, (1.68 ± 0.38) m and (1.28 ± 0.31) m, respectively, *p*-value < 0.0005 (3 stars in Figure 6);

Figures 7–9 describe the behavior of the two systems *adapt-after-evo* (on the left column) and *adapt-in-evo* (on the right one) in the three different friction conditions with the terrain (Figure 7 is high friction, Figure 8 is medium friction and Figure 9 is low friction). To analyze data from a representative experiment, the plots (Figures 7–9) include the behavior of one of the ten reiterations of the robotic individual whose performance, in covered distance *D*, is the closest to the average behavior among all the individuals in the two control cases *adapt-after-evo* and *adapt-in-evo*, for all the 3 levels of friction. This selected agent has a noise injected in the encoder which is 2% of its total motor signal, while its joints damping coefficient is 0.19 $\frac{Ns}{m}$.

In all three cases (Figures 7–9), subplots (a) (*adapt-after-evo*) and (e) (*adapt-in-evo*) highlight that during the first minute of simulation, the position errors at the joint level are decreasing, even if the experimental conditions (robotic model and robot-ground friction coefficient) are changed compared to the initial simulation constraints, where the locomotion patterns have been found. The error for the system *adapt-in-evo* (right column) is always smaller than for the other system *adapt-after-evo* (left column), observing both its mean and standard deviation across the four legs. In the three different robot-ground interactions (Figures 7–9), the Root Mean Square Error (RMSE) in the following of the desired joint trajectories is shown in Table 3.

**Table 3:** Root Mean Square Error in following the desired locomotion trajectories for the representative Fable robot individuals.

**Friction coefficient**	**Adapt-after-evo**	**Adapt-in-evo**
High	0.056 rad	0.035 rad
Medium	0.039 rad	0.033 rad
Low	0.044 rad	0.041 rad

*These values are related to Figures 7A,E, 8A,E, 9A,E*.

The contributions of the different modules of the controller architecture (subplots b and f) show the same trend as in Figure 5; after a few seconds after the beginning of the simulation, *u-lwpr* becomes predominant and *u-pl* learns the *u-fb* and they together decrease their contributions along the simulation.

The most significant differences between the behavior of two compared systems *adapt-after-evo* and *adapt-in-evo* without disturbances (Figure 5) and that when the dynamics of the experiments have been changed (Figures 7–9), can be observed in subplots (c, d, g, h). At joints level (Figures 7C,G, 8C,G, 9C,G), the performances of the two systems *adapt-after-evo* and *adapt-in-evo* demonstrate a less stable behavior if compared to the same subplots (c) and (g) in Figure 5. The trend of the joins trajectories still converges to the limit cycle obtained by the position references, which is indicated in pink, and to the periodic shape got in the last 10 s of simulation for the same system without disturbances. However, lower the friction coefficient value, longer the time the systems take to converge to the desired periodic behavior (Figures 7–9). It is also relevant to point out that the entropy of the joint trajectories increases in inverse proportion to the static friction coefficient of friction with the ground (the minimum tested static friction coefficient is showed in Figure 9).

Eventually, a meaningful index of the difference in the stability response of the two systems *adapt-after-evo* and *adapt-in-evo* is the plot showing the dynamics of the Center of Mass (CoM) of the robot (d, h). Here, the stability region in the no disturbances case is represented in red, while the behavior for the affected systems is in the remaining color gradient timeline (Figures 7, 8, 9D,H). In all the three figures (Figures 7, 8), the behavior of the *adapt-in-evo* agent (on the right) is confined in a region of the phase space which is very close to region covered by the dynamics of the same system without disturbances (in red in subplots d and h). Instead, the dynamics of the center of mass of the *adapt-after-evo* experiments (on the left column in Figures 7–9) are always more unstable than its equivalent *adapt-in-evo* (Figures 7–9), meaning that the adaptability, brought by the cerebellar inspired module, as a control feature during the evolutionary exploration for effective locomotion trajectories, contributes to discovery more flexible robotic locomotion patterns.

### 3.3. Dynamically Changing Experimental Set-Up

After testing the control architecture with a set of simulated Fable robots with different dynamical characteristics and friction interactions with the environment, further experiments are performed. This set of tests has been carried out to compare the performances of the two systems with respect to scenarios in which the interaction with the environment changes dynamically. In this case, the static friction coefficient is changed during the experiment, respectively, at 50 and 100 s from the beginning of the simulation and the simulation lasts 2 min in total.

For these experiments, the same representative individual we choose for designing the previous plots (2% of the motor signal as noise in the encoders and 0.19 $\frac{Ns}{m}$ of joints damping coefficient) is tested for the dynamically changing set-up, and the simulations are run 5 times per type of controller (*adapt-after-evo* and *adapt-in-evo*).

Concerning the task space, the average, among the 5 trials, of the distance covered by the robot, from 50 to 120 s of simulation, is 6.18 m for the *adapt-after-evo* and 10.28 m for the *adapt-in-evo*, respectively, with standard deviation 2.25 and 2.40 m.

**Figure 10:**
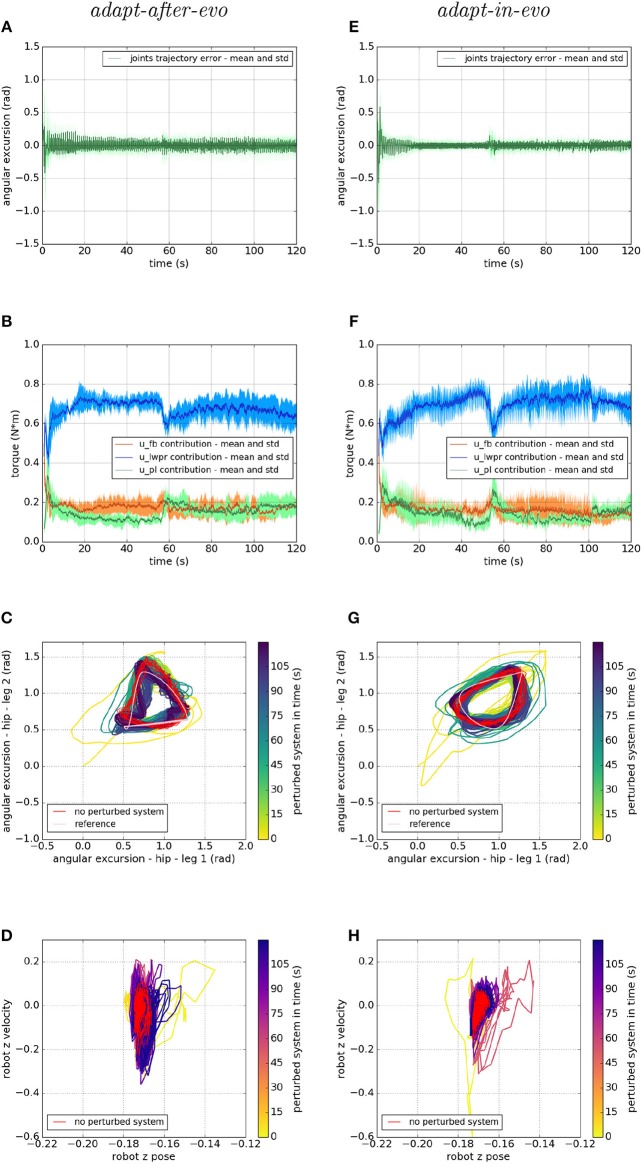
Locomotion performance and characterization of the two systems; on the left the *adapt-after-evo* system and, on the right, the *adapt-in-evo* one, with a friction coefficient that has been changed from 1 to 0 at 50 s and from 0 to 0.5 at 100 s. **(A,E)** Represent the mean and the standard deviation of the position error of the four legs joints and **(B,F)** instead describe the mean and the standard deviation of the contribution ratio of the different modules of the control architecture. **(C,G)** Describe the periodic behavior relation between the actual joint trajectories of leg 1 and leg 2 compared to their reference values, in pink, and to the behavior of the no perturbed system, in red (among the other pairs of legs, the relation is periodic in a comparable way). Eventually, **(D,H)** represent the dynamics of the CoM of the robot, on the vertical axis to the ground, compared the same CoM dynamics when the system is not perturbed (in red).

In Figure 10, we show the response of the two systems *adapt-after-evo*, on the left, and *adapt-in-evo*, on the right, when the friction coefficient is dynamically changed during the simulation. As explained in section 3.2, the initial static friction coefficient is 1, the maximum value allowed in the Gazebo simulator and then it is decreased to 0, its minimum, around 50 s from the beginning of the simulation, and increase again to 0.5 at 100 s. In Figure 10 the same graphs, as for the previous experiments, are shown. In subplots (a) and (e) the mean and standard deviation of the legs are shown. A fast spike is visible around 50 s of simulation when the interaction with the environment is changed, but then the position error decreases again and a slight change in the graph is also visible around 100 s when the friction is changed again. Both systems *adapt-after-evo* and *adapt-in-evo* reject the disturbance given by changing the static friction coefficient. Also, in this case, the assessment of the advantage brought by the *adapt-in-evo* controller is quantitatively proved by the RMSE which is 0.05 radian in the *adapt-after-evo* and 0.04 radian in the *adapt-in-evo* one.

In Figures 10B,F, it is clear that around 50 s of the simulation, an unexpected change perturbs the system and the *u-lwpr* and *u-pl* need to learn again the model of the interaction among robot and ground. The second change in the static friction coefficient is lightly visible around 100 s from the beginning of the simulation.

In conclusion, in the Figures 10C,D,G,H, the difference in the rejection of the disturbances among the two systems *adapt-after-evo* and *adapt-in-evo*, is more evident. In fact, after the second 50 of simulation, the *adapt-after-evo* is not able to completely recover from the disturbance. In fact, the last seconds of simulation (in dark blue) are slightly different from the behavior of the no-perturbed system (in red). This happens both at joint level in Figure 10C and at the task level in Figure 10D. On the contrary, the *adapt-in-evo* system feels the change in the interaction with the environment, but it can return to a state of the system which is closer to the initial one whose response is highlighted in red. The temporary divergence of the behavior of the system is visible around second 50 either in Figure 10G, in light green, and in Figure 10H, in pink. In these final subplots (c, e, g, h), the second change in the static friction coefficient does not have an evident impact, either in the *adapt-after-evo* and in the *adapt-in-evo* case. A significant divergence in the locomotion stability of the system is visible just in the dynamics of the CoM of the *adapt-after-evo* system in Figure 10D.

## 4. Discussion

For the first time, taking inspiration from nature, the proposed research uses robotics to suggest the advantages and benefits of employing adaptive controllers in conjunction with optimization strategies, such as evolutionary algorithms. For this purpose, a new bio-inspired approach to control robotic locomotion is presented. The control design is based on neurophysiological evidences concerning a simplified model of the neural control in the locomotion of quadruped animals. In the proposed control architecture, the trajectory planner is a CPG-inspired system of equations and the motion controller is composed of a PID and a bio-inspired algorithm, whose weights are changing on-line with the simulation time. This latter part of the architecture models the adaptive role of the Cerebellar-inspired circuit in the locomotion of vertebrates which encodes information about the inverse dynamic model of the quadruped.

The main contribution of the paper is investigating the advantages of using a learning control module during the optimization of the locomotion patterns for a quadruped robot rather than employ it when the optimal locomotion patterns have already been found (as it is usually done in already existing approaches, Urbain et al., 2018; Vandesompele et al., 2019). This idea comes from nature since evolution has always been acting on plastic and learning systems. The research aims to investigate if the solutions found out by the evolution-inspired algorithm are statistically better when a learning module is included in the controller, during the evolution. The presented approach shows the advantages of this optimization procedure for quadruped robotic locomotion both in the task and in the joint space. The distance covered by the robot is greater when the learning module is involved in the genetic optimization process and, the position error of the joints is smaller.

These results are also reflected in new experiments when the robot dynamic characteristics are changed, and some noise is injected in the robot encoders. The preponderance of the *adapt-in-evo* solution has been generalized by running other experiments with a different robot-environment interaction, which allows to infer the crossing of the reality gap. Further, the robot-ground interaction has also been dynamically changed during the experiments, assessing the potential of the *adapt-in-evo* approach in readjusting to different experimental constraints even though learning stability has already been reached by the cerebellar inspired module. The results show that the inclusion of the cerebellar-inspired control in the process of optimization of the locomotion trajectories allow a maximization of the synergy between the CPG-inspired trajectory planner and the adaptive cerebellar controller. The best patterns, which emerge during the previously explained synergy, are more robust. Even when the experimental conditions change, in the dynamics of the robot and in its interaction with the environment, before or during the experiments, the locomotion preserves more stability both at joint and task level.

In conclusion, further investigations can be done by testing the architecture on the real Fable robot since the conducted experiments aimed at proving the suitability of employing the same controller in real scenarios. In fact, the results show that both control strategies, *adapt-after-evo* and *adapt-in-evo*, are robust enough to work, without changing parameters, in unexpected conditions such as noisy sensors or slippery terrains (also applied in the same experiment).

## Data Availability

The datasets generated for this study are available on request to the corresponding author.

## Author Contributions

The bio-inspired control architecture was primarily developed by EM and ST. The use of the evolution-based approach was mainly handled by EM, GU, AV, and JD. EM, LV, UA, and MC worked on the implementation of the experiment. EM, AS, and EF statistically analyzed and interpreted the data. EM, LV, ST, EF, and CL wrote and reviewed the manuscript. All authors read and approved the final manuscript.

### Conflict of Interest Statement

The authors declare that the research was conducted in the absence of any commercial or financial relationships that could be construed as a potential conflict of interest.
